# Huangqi Shengmai Yin Ameliorates Myocardial Fibrosis by Activating Sirtuin3 and Inhibiting TGF-β/Smad Pathway

**DOI:** 10.3389/fphar.2021.722530

**Published:** 2021-08-13

**Authors:** Jianheng Pan, Zhanhong Cao, Chunqiu Fang, Yuting Lei, Jiaming Sun, Xiaowei Huang, Dong Han

**Affiliations:** ^1^ Department of Pharmacy, Changchun University of Chinese Medicine, Changchun, China; ^2^ Jilin Ginseng Academy, Changchun University of Chinese Medicine, Changchun, China

**Keywords:** Huangqi Shengmai Yin, myocardial fibrosis, isoprenaline, sirtuin 3, TGF-β/Smad pathway

## Abstract

Myocardial fibrosis (MF) is an important pathological process in which a variety of cardiovascular diseases transform into heart failure. The main manifestation of MF is the excessive deposition of collagen in the myocardium. Here, we explored whether Huangqi Shengmai Yin (HSY) can inhibit isoprenaline (ISO)-induced myocardial collagen deposition in rats, thereby reducing the cardiac dysfunction caused by MF. The results of echocardiography showed that HSY upregulated fractional shortening and ejection fraction, and reduced the left ventricular systolic dysfunction in the rats with MF. Pathological results showed that HSY protected myocardium, inhibited apoptosis, and effectively reduced collagen deposition. HSY also inhibited the expression of collagen I and III and α-smooth muscle actin (α-SMA) in the heart tissue. HSY increased the expression of Sirtuin 3 (Sirt3) and inhibited the protein levels of the components in the transforming growth factor-β (TGF-β)/Smad pathway. At the same time, it also regulated the expression of related proteins in the matrix metalloproteinases family. In summary, HSY played a therapeutic role in rats with ISO-induced MF by protecting myocardium and inhibiting collagen deposition. Therefore, HSY is a potential therapeutic agent for ameliorating MF.

## Introduction

Despite the increasing development of medicine and living standards, cardiovascular diseases are still currently the main cause of death (Oka et al., 2014). Many diseases, such as dilated cardiomyopathies, diabetic cardiomyopathies, and acute myocardial infarctions, are associated with myocardial fibrosis (MF) (McDonald et al., 2018; Yuan et al., 2018; Zhang et al., 2018). Delaying the development of MF is thus an effective strategy to reduce the risk of death from cardiovascular disease.

MF is caused by excessive accumulation of extracellular matrix proteins, which contributes to systolic and diastolic dysfunction, ultimately leading to the heart failure. It has been reported that the Renin-Angiotensin-Aldosterone System (RAAS) is involved in MF through transforming growth factor-β (TGF-β) (Gourdie et al., 2016), angiotensin II(Ang II) (He and Ou 2020), and other factors. After myocardial infarction occurs in the body, the process of MF is activated, a large amount of collagen is secreted, and scar tissue is formed by deposition. On the one hand, the formation of scar tissue ensures the integrity of the myocardial tissue; however, on the other hand, the scar tissue that is formed due to excessive MF reduces myocardial elasticity, leading to a systolic and diastolic dysfunction (Leask 2015). At the same time, some related cytokines that promotes cardiomyocyte hypertrophy are also secreted, resulting in heart failure and even death (Mohammed et al., 2015; Stuart et al., 2016). Therefore, many researchers have been exploring safe and effective methods to ameliorate MF. In recent years, many studies have shown that Traditional Chinese medicine (TCM) has a significant effect in the alleviation of MF (Lv et al., 2020).

Huangqi Shengmai Yin (HSY) is a Chinese medicine compound prescriptions which is evolved from the basic prescription of Sheng mai power and composed of *Astragalus mongholicus* Bunge, *Codonopsis pilosula* (Franch.) Nannf., *Ophiopogon japonicus* (Thunb.) Ker Gawl., and *Schisandra chinensis* (Turcz.) Baill. (Table 1). HSY is traditionally used to treat cardiovascular diseases, respiratory diseases and improve immunity (Liu et al., 2009). Modern pharmacological studies have shown that HSY has pharmacological effects such as dilating blood vessels, increasing myocardial contractility and anti-virus. Clinically, HSY is mainly used to treat viral myocarditis, coronary heart disease and heart failure (Jiang et al., 2021). In addition, some studies have shown HSY can alleviate radiation-induced MF (Gu et al., 2019). However, the protective effects of HSY against MF after myocardial infarction have not yet been reported. In this study, we aimed to examine the hypothesis that HSY exerts its cardioprotective effect by inhibiting collagen deposition in a rat model of isoprenaline (ISO)-induced MF. Considering the fact that captopril, an angiotensin-converting enzyme inhibitor, has antifibrotic activity by diminishing MF associated with enhanced collagen degradation, the drug was used as a positive control in this study (Brilla et al., 2003; Guo et al., 2010; Wang, Shen, et al., 2019).

**TABLE 1:** Composition of HSY.

Component	Family	Concentrations (g/100 ml)
*Astragalus mongholicus* Bunge	Leguminosae	30
*Codonopsis pilosula* (Franch.) Nannf	Campanulaceae	20
*Ophiopogon japonicus* (Thunb.) Ker Gawl	Liliaceae	20
*Schisandra chinensis* (Turcz.) Baill	Magnoliaceae	10

## Materials and Methods

### Preparation of Huangqi Shengmai Yin

The HSY (batch no. 190916) was purchased from the Nan Chang Ji Sheng Pharmaceutical Factory (Nanchang, China). HSY (GB (2014)Z-0058)) was routinely prepared according to Chinese standard (No. WS3-B-3102-98-1). *Codonopsis pilosula* (Franch.) Nannf. (200 g), *Ophiopogon japonicus* (Thunb.) Ker Gawl. (200 g) and *Schisandra chinensis* (Turcz.) Baill. (100 g) were extracted twice by boiling water, and the two decoctions were combined, filtered, concentrated, and precipitated by ethanol to remove the impurities. Ethanol was recovered from the supernatant by evaporation, and then the supernatant was suspended in water, and medicinal charcoal was added, and then the filtrate was filtered to form extract A. *Astragalus mongholicus* Bunge (300 g) was extracted by boiling water twice, and the two decoctions were combined, then filtered, concentrated, and then precipitated by ethanol. The precipitate was dissolved with ethanol, filtered, and ethanol was removed. Then it was combined with the above-mentioned extract A. Steviosin (0.2 g) and sodium benzoate(3 g) were added, and water was added to 1000ml, and then the pH was adjusted, stirred, sealed, and sterilized. Finally, HSY was obtained. In the final preparation solution, the concentration of *Codonopsis pilosula* (Franch.) Nannf. was 0.2 g/ml, the concentration of *Ophiopogon japonicus* (Thunb.) Ker Gawl. was 0.2 g/ml, the concentration of *Schisandra chinensis* (Turcz.) Baill. was 0.1 g/ml, the concentration of *Astragalus mongholicus* Bunge was 0.3 g/ml.

### Reagents and Drugs

The ISO was obtained from Sigma-Aldrich (St. Louis, MO, United States). The captopril was purchased from the PURCON Pharmaceutical Factory (Shanghai, China). The creatine kinase (CK), hydroxyproline (HYP), and lactate dehydrogenase (LDH) kits were purchased from Nanjing Jiancheng Bioengineering Institute (Nanjing, China). The creatine kinase-MB (CK-MB), procollagen type I carboxy-terminal peptide (PICP), and type I collagen carboxyl terminal peptide (ICTP) ELISA kits were purchased from Meimian Biotechnology Institute (Shenyang, China). The primary antibodies against Sirt3, Smad2, Smad4, Smad7, GAPDH, Matrix Metalloproteinase (MMP) 2, and MMP9 were purchased from Proteintech Group, Inc. (Wuhan, China). The primary antibodies against TGF-β and Smad3 were purchased from Bioss Co. (Beijing, China). The primary antibody against Tissue Inhibitors of Metalloproteinase (TIMP)-1 was purchased from Shanghai Bowan Biotechnology Co., Ltd. (Shanghai, China).

### UPLC-ESIQ-Exactive Orbitrap/MS Analysis

The chemical compositions of HSY were identified by UPLC-ESIQ-Exactive Orbitrap/MS according to the previous study (Xin and Bai 2021). The chromatographic method was achieved adopting ACQUTIY UPLC BEH C18 (50 mm × 2.1 mm × 1.7 μm) as a stationary phase and 0.1% formic acid (A)/ 100% acetonitrile (B) as mobile phase with gradient elution at a constant flow rate of 0.25 ml/min. The elution order was as follows: maintained with 10% B in 3 min, linear gradient from 10% B to 30% B in 3 min, 30% B to 50% B in 3 min, 50% B to 70% B in 3 min, 70% B to 80% B in 3 min, 80% B to 95% B in 3 min, maintained with 95% B in 4 min, 95% B to 10% B in 0.1 min, maintained with 10% B in 2.9 min. Mass spectrometric detection was carried out on a Q-Exactive quadrupole electrostatic field orbitrap high resolution mass spectrometry (Thermo Fisher Scientific, MA, United States ). The electrospray ionization source in both positive (ESI+) and negative (ESI−)ion modes was used with scanning range of m/z 100∼1,500. The MS source parameters were set as follows: sheath gas flow of 20 arbitrary units, aux gas (Nitrogen) flow of 5.7 arbitrary units. The capillary voltage was set to +3.5 kV and −2.8kV at the capillary temperature of 200°C and aux gas heater temperature of 350°C. The scan mode was Full MS, of which the resolution was 70,000 (Full MS). Data are recorded and analyzed using the Xcalibur software (Version 2.2.42, Thermo Fisher Scientific, MA, United States).

### Experimental Animals and Protocols

Male Sprague-Dawley rats (weighing 200–230 g) were purchased from Changchun Yisi Laboratory Animal Technology Co., Ltd. (Jilin, China). All the animals were housed in a room according to a 12 h light/dark cycle and had free access to a standard diet and drinking water. Fifty rats were assigned randomly into five groups: the control group (Con, *n* = 10), ISO group (ISO, *n* = 10), captopril group (Cap, *n* = 10, 13.5 mg/kg/d), low-dose HSY group (HSY-L, *n* = 10, 2.7 ml/kg/d), and high-dose HSY group (HSY-H, *n* = 10, 5.4 ml/kg/d). Based on the doses used in previous studies (Wang, Yu, et al., 2019), ISO (5 mg/kg, IH.) was injected once daily for 7 days to induce MF. The rats were injected with the same volume of normal saline in the control group. The dose and duration of use of HSY and captopril were calculated based on the equivalent dose ratio (approximately 6.3) between humans and rats in terms of body surface area. The dose of HSY-L group and captopril group were equivalent to human dose, and the dose of HSY-H group was twice the dose of HSY-L group. The rats in the HSY and captopril groups were given HSY and captopril respectively for 4 weeks, while the rats were administered the same volume of distilled water in the control and ISO groups. Four weeks later, all rats were performed by echocardiography. All the experiments were approved by the Experimental Animal Ethics Committee of Changchun University of Chinese Medicine (Approval Number: 2020319) and were performed in accordance with the guidelines of the National Institute of Health Guide for the Care and Use of Laboratory Animals.

### Echocardiography Measurements and Determination of Serum Biomarkers

At the end of the 4^th^ week, the rats in each group were anesthetized with 40 mg/kg of pentobarbital, and the chest hair was removed with an electric hair shaver. Cardiac function was evaluated by echocardiography with a vivid-I ultrasound machine (General Electric Company, America) with a 10* *s sensor. The ejection fraction (EF) and fractional shortening (FS) were measured. The blood was collected from the abdominal aorta and centrifuged at 3,000 rpm/min (4°C) for 15 min. The serum was then collected and stored at −80°C until analysis. The serum levels of CK, LDH, and HYP were measured according to the manufacturer’s instructions. In addition, the serum CK-MB, PICP and ICTP were quantified by ELISA kit according to the manufacturer’s instructions.

### Hematoxylin-Eosin Staining and Masson Staining

The heart was embedded in paraffin with a section thickness of 5 μm. Hematoxylin-eosin (HE) was performed after routine dewaxing and hydration. The morphological characteristics of the injured myocardial tissue were observed by HE staining.

The progress degree of MF was observed by Masson staining. The sections were incubated with Masson’s trichrome stain following routine methods. The cardiomyocytes were visualized as red-stained areas and collagen was visualized as blue-stained areas. The collagen volume fraction (CVF) of the cardiac tissue was quantified using Image J software (National Institutes of Health, America). The formula used to calculate the CVF was myocardial interstitial collagen area/total visual field area.

### Immunohistochemistry

The paraffin sections were deparaffinized in water. The endogenous peroxidase blocker was dropped onto the tissue, incubated for 10 min, and rinsed with double-distilled water. The antigen was then repaired with microwave heating. The sections were blocked with BSA for 30 min. Subsequently, the sections were incubated with collagen I, collagen III, and α-Smooth Muscle Actin (α-SMA) (1:100) antibodies at 4°C overnight. Goat anti-rabbit/mouse IgG was then added dropwise, and the sections were incubated for 30 min. After 30 min with streptavidin/peroxidase complex, 3,3′-diaminobenzidine hydrochloride color culture was added dropwise to the slice. Subsequently, hematoxylin restaining was performed for 2 min. The histochemical score (H-Score) was calculated using the Image-Pro Plus software 6.0 (Media Cybernetics, Inc. America) according to the equation: H-Score = Ratio of Strong-Positive*3 + Ratio of Moderate-Positive*2 + Ratio of Weak-Positive*3.

### Western Blotting

The total protein from the left ventricular tissue was extracted by a RIPA lysis buffer (Beijing Ding Guo Chang Sheng Biotechnology Co., Ltd. China), and protein concentration is determined via bicinchoninic acid (BCA) Protein Assay kit. Equal amounts of protein were separated using SDS-PAGE. Proteins on the gel were transferred to a polyvinylidene fluoride (PVDF) membrane (Millipore Co., Ltd. NJ, United States ) and blocked with 5% non-fat milk for 2 h. The membrane was then incubated with the primary antibodies overnight at 4°C. The primary antibodies were: anti-TGF-β (1:500), anti-Sirt3 (1:500), anti-Smad2 (1:1,000), anti-Smad3 (1:500), anti-Smad4 (1:500), anti-Smad7 (1:500), anti-MMP2 (1:500), anti-MMP9 (1:500), anti-TIMP1 (1:500), and anti-GAPDH (1:20000). After the membranes were washed, the membranes were incubated with HRP-conjugated secondary antibodies, and target proteins were developed by an enhanced chemiluminescence imager. Image J software was used to analyze the band intensity of the immune response quantitatively and to calculate the content of the target protein.

### Statistical Analysis

All data have been tested for normality and presented as mean ± SD and analyzed using GraphPad Prism 8 software (GraphPad Software, San Diego, CA, United States ). Student’s t-tests were used to compare the differences between two groups. One way analysis of variance (ANOVA) followed by Tukey’s multiple comparison test were used to compare more than two groups. *p* < 0.05 was considered to be statistically significant.

## Results

### Identification of Main Bioactive Compounds in Huangqi Shengmai Yinby UPLC-ESIQ-Exactive Orbitrap/MS Analysis

**FIGURE 1:**
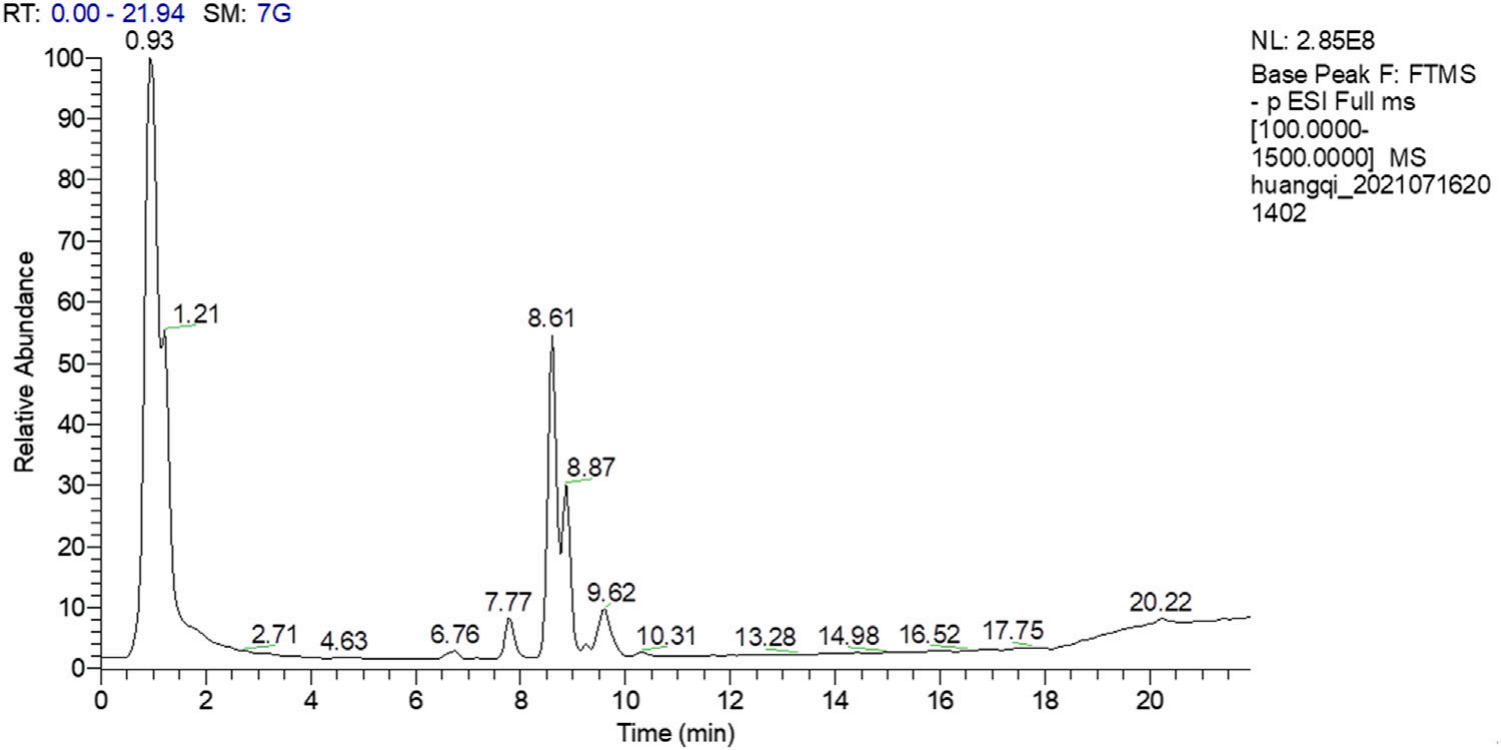
Total ion chromatogram of HSY by UPLC-ESIQ-Exactive Orbitrap/MS.

A total of 18 chemical compounds were identified in HSY, namely calycosin-7-glucoside, lobetyolin, ononin, isomucronulatol 7-O-glucoside, isomucronulatol, 9-hydroxy-10,12-octadecadienoic acid, 7-hydroxy-3-(3-hydroxy-4-methoxyphenyl)-4H-chromen-4-one, 9,12,13-trihydroxy-10,15-octadecadienoic acid, schisandronic acid, astragaloside IV, 9,10,13-trihydroxy-11-octadecenoic acid, formononetin, ophiopogonin C, astragaloside II, astragaloside I, gomisin A, ophiopogonin D and 9,10-dihydroxy-12-octadecenoic acid (Figure 1; Table 2).

**TABLE 2:** The chemical components identified from HSY.

NO	RT (min)	Formula	Molecular weight	ppm	Compounds
1	6.62	C_22_H_22_O_10_	446.40408	2.17	Calycosin-7-glucoside
2	7.77	C_20_H_28_O_8_	396.43152	1.85	Lobetyolin
3	8.01	C_22_H_22_O_9_	430.40468	2.28	Ononin
4	8.43	C_23_H_28_O_10_	464.46242	2.69	Isomucronulatol 7-O-Glucoside
5	8.48	C_17_H_18_O_5_	302.32182	3.06	Isomucronulatol
6	8.59	C_18_H_32_O_3_	296.44488	3.24	9-hydroxy-10,12-octadecadienoic acid
7	8.64	C_16_H_12_O_5_	284.26348	2.61	7-hydroxy-3-(3-hydroxy-4-methoxyphenyl)-4H-chromen-4-one
8	9.38	C_18_H_32_O_5_	328.44368	3.42	9,12,13-Trihydroxy-10,15-octadecadienoic acid
9	9.70	C_30_H_46_O_3_	454.68444	-3.10	Schisandronic acid
10	9.73	C_41_H_68_O_14_	784.97022	2.54	Astragaloside IV
11	9.78	C_18_H_34_O_5_	330.45956	3.28	9,10,13-Trihydroxy-11-Octadecenoic Acid
12	10.18	C_16_H_12_O_4_	268.26408	3.99	Formononetin
13	10.20	C_44_H_70_O_18_	887.0158	1.30	Ophiopogonin C
14	10.95	C_43_H_70_O_15_	827.0069	2.57	Astragaloside II
15	11.72	C_45_H_72_O_16_	869.04358	3.52	Astragaloside I
16	12.04	C_23_H_28_O_7_	416.46422	-3.65	Gomisin A
17	12.59	C_44_H_70_O_16_	855.017	2.68	Ophiopogonin D
18	12.85	C_18_H_34_O_4_	314.46016	4.13	9,10-Dihydroxy-12-Octadecenoic Acid

### Huangqi Shengmai Yin Prevents Cardiac Dysfunction in the Rat Myocardial Fibrosis Model Induced by Isoprenaline

**FIGURE 2:**
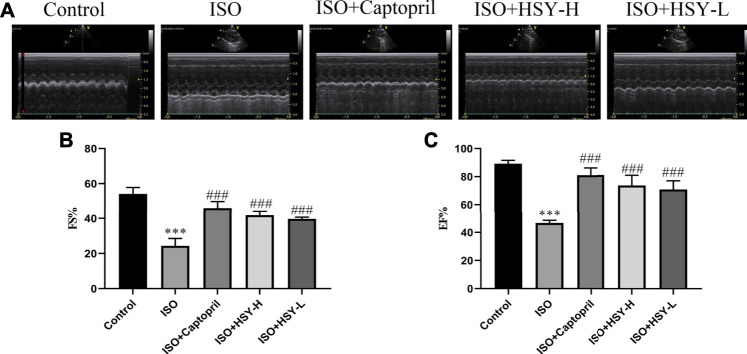
The effect of HSY on the echocardiography of ISO-induced MF in rats. **(A)** Representative images of ultrasound in different groups. **(B–C)** Fractional shortening (FS) and ejection fraction (EF) was used to evaluate left ventricular systolic function. (*n* = 6). Data are shown as the mean ± SD. Significance: ^***^ *p* < 0.005 vs control group; ^###^<0.005 vs ISO group.

We used echocardiogram to determine the cardiac function effect of HSY on rats. As shown in Figure 2, the EF and FS of rats were reduced significantly in the ISO group, indicating severe ventricular systolic dysfunction and reduced cardiac output. After 4 weeks of treatment, the EF and FS of rats were improved in the HSY groups or captopril group.

### Effects of Huangqi Shengmai Yin on Myocardial Injury on the Rat Myocardial Fibrosis Model Induced by Isoprenaline

**FIGURE 3:**
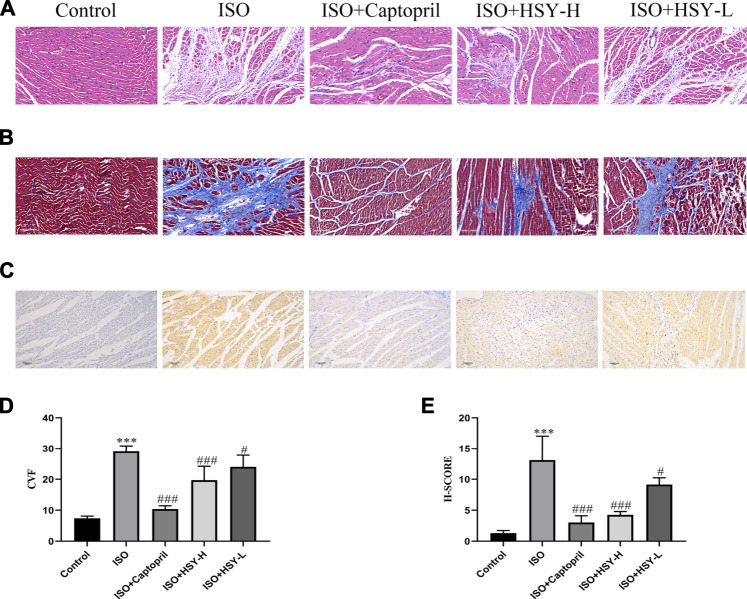
Effect of HSY on histopathological changes in rat heart tissue. **(A)** Representative images of HE staining of left ventricular tissue in different groups. **(B)** Representative images of Masson trichrome staining of left ventricular tissue in different groups. **(C)** Representative images of Tunel staining of left ventricular tissue in different groups. **(D)** The quantitative analyses of area percentage of collagen deposition (*n* = 6). **(E)** The H-SCORE of Tunel staining of different groups (*n* = 6). Data are shown as the mean ± SD. Significance: ^***^ *p* < 0.005 vs control group; ^#^ *p* < 0.05 vs ISO groups; ^##^ *p* < 0.01 vs ISO group; ^###^ *p* < 0.005 vs ISO group.

**FIGURE 4:**
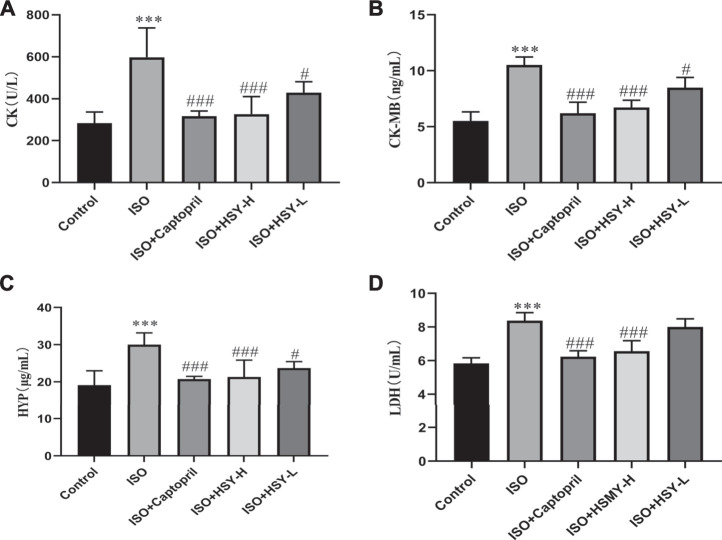
HSY significantly reduced myocardial injury in ISO-induced cardiac fibrosis rats. **(A–D)** Serum CK, CK-MB, HYP and LDH of each group was determined using kits. Data are shown as the mean ± SD. Significance: ^***^ *p* < 0.005 vs control group; ^#^ *p* < 0.05 vs ISO groups; ^###^ *p* < 0.005 vs ISO group.

Pictures of hematoxylin-eosin (HE)-stained myocardial tissue are shown in Figure 3A. Compared with the control group, ISO group showed severe myocardial structural alterations, including inflammatory infiltration, and nuclear lysis. There were less inflammatory infiltration and nuclear lysis of the cardiomyocytes in the HSY-L group. There was a significant improvement in the myocardium in the HSY-H or captopril group, with a neat arrangement and only a small number of inflammatory infiltrates. The myocardial enzymes were also evaluated (Figure 4). The CK, CK-MB, LDH, and HYP levels of rats in the ISO group were significantly higher than those in the control group. With HSY or captopril intervention, the above myocardial enzymes were reduced significantly.

Through TUNEL staining of the myocardial tissue of each group, there are a large number of apoptotic cells in the ISO group (Figures 3C,E). After the intervention of HSY or captopril, the number of apoptotic cells was reduced significantly, which suggested that HSY or captopril could inhibit the apoptosis of cardiomyocytes.

### Effect of Huangqi Shengmai Yin on the Expression of Collagen and α-Smooth Muscle Actin in the Rat Myocardial Fibrosis Model Induced by Isoprenaline

**FIGURE 5:**
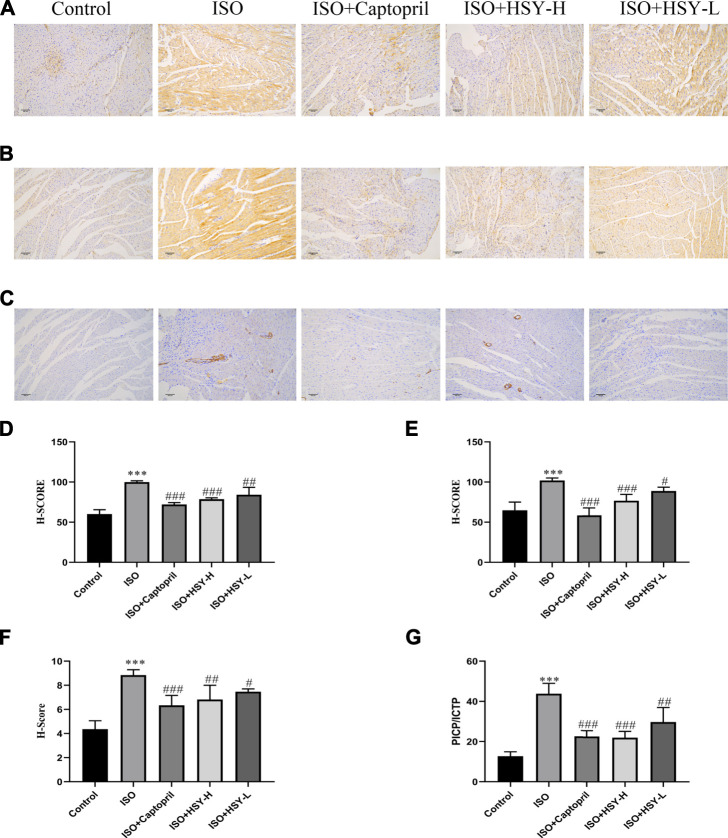
The effect of HSY on the expression of collagen and α-SMA in each group. **(A–C)** Representative images of different groups of collagen I, III and α-SMA immunohistochemistry. **(D–F)** The H-SCORE of collagen I, III and α-SMA in different groups (*n* = 6). **(G)** The ratio of the content of PICP and ICTP in each group of serum. Data are shown as the mean ± SD. Significance: ^***^ *p* < 0.005 vs control group; ^#^ *p* < 0.05 vs ISO groups; ^##^ *p* < 0.01 vs ISO group; ^###^ *p* < 0.005 vs ISO group.

Masson staining was used to evaluate the amount of collagen deposition in the myocardium (Figures 3B,D). The results showed that with ISO intervention, there was a large amount of blue collagen deposition in the myocardium. HSY or captopril reduced the collagen deposition in the myocardium, significantly. The ELISA results showed that the ratio of PICP/ICTP was increased significantly in the ISO group, while the ratio of PICP/ICTP was decreased in the HSY groups or captopril group. (Figure 5G).

To further confirm the inhibitory effect of HSY on the MF induced by ISO, immunohistochemical staining was used to evaluate the content of collagen I and collagen III, which are the two main components of the extracellular matrix. The α-SMA expression was also evaluated with immunohistochemistry. The immunohistochemical results showed that compared with the control group, the expression of myocardial collagen I and collagen III were increased significantly in myocardial tissue in the ISO group (Figures 5A,B,D,E). Compared with the ISO group, the expression of myocardial collagen I and collagen III was decreased with HSY or captopril intervention. The immunohistochemical results of the α-SMA showed that compared with the control group, more brown particles were seen in the ISO group, while that HSY or captopril reduced the intensity of the positive staining (Figures 5C,F).

### Effect of Huangqi Shengmai Yin on Sirtuin 3, Transforming Growth Factor-β/Smad Signaling Pathway and Matrix Metalloproteinase-Related Proteins Expression

**FIGURE 6:**
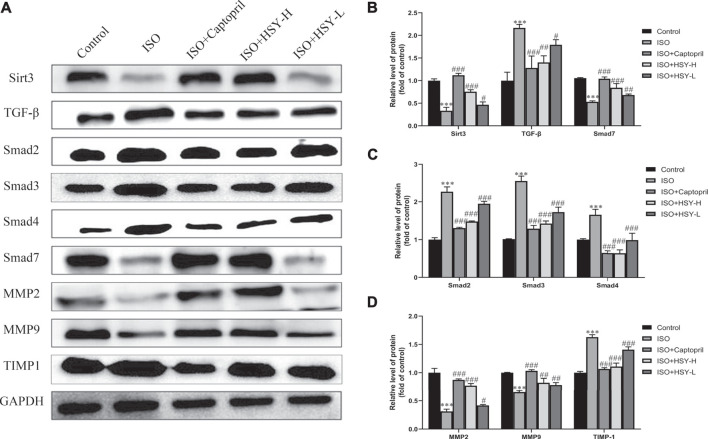
Effect of HSY on the Sirt3/TGF-β/Smad signaling pathway in rats. **(A)** The level of Sirt3/TGF-β/Smad signaling pathway and MMPs in each group.**(B–D)** Quantified protein bands of Sirt3/TGF-β/Smad signaling pathway and MMPs with GAPDH as control. Data are shown as the mean ± SD. Significance: ^*^ *p* < 0.05 vs control group; ^***^ *p* < 0.005 vs control group; ^#^ *p* < 0.05 vs ISO group; ^##^ *p* < 0.01 vs ISO group; ^###^ *p* < 0.005 vs ISO group.

In order to clarify the molecular mechanisms of HSY in the treatment of ISO-induced MF, western blot experiments were performed to detect the Sirt3, TGF-β/Smad signaling pathways, and the related protein expressions of MMPs (Figure 6A). As shown in Figures 6B–D, compared with the control group, the expressions of TGF-β, Smad2, 3, and 4 were increased, while the expressions of Sirt3 and Smad7 were decreased in the ISO group. Among the MMP-related proteins, the expressions of MMP2 and 9 were decreased, while the expression of TIMP-1 was increased in the ISO group. Compared with the ISO group, HSY or captopril upregulated the expressions of Sirt3 and Smad7 and downregulated the expressions of TGF-β and Smad2, 3, and 4. In addition, HSY or captopril also upregulated the protein expressions of MMP2 and MMP9 in the MMP family and inhibited the expression of TIMP-1.

## Discussion

With the intensification of global aging, cardiovascular diseases have become the main cause of death (Arnett et al., 2019). MF is an important pathological feature of cardiovascular diseases because it can lead to ventricular remodeling (Hughes et al., 2019). Many cardiovascular diseases, such as hypertension, myocarditis, and myocardial infarction, can cause MF. (Dixon and Spinale 2010; Masci et al., 2013). The effective prevention of MF is of great significance for prolonging lives of patients with cardiovascular diseases.

MF is an abnormal deposition of the extracellular matrix (ECM) in the myocardium, which is characterized by collagen deposition increasing in the gap. Collagen I and III are the main components of the cardiac ECM (Essick and Sam 2011; Li et al., 2018). This excessive deposition of collagen reduces the elasticity of the myocardium, causing disorders in the diastolic and systolic function of the heart leading to heart failure and even sudden cardiac death (Czubryt 2012; Raman et al., 2019). We assessed the cardiac function of the rats in each group by echocardiography and found the rats developed severe heart failure, and their EF and FS decreased significantly, suggesting that the rats had left ventricular systolic dysfunction in the ISO group. Compared with the ISO group, the EF and FS of the rats were increased in the HSY-H group and HSY-L group, indicating that the left ventricular systolic disorder was relieved, which suggested that HSY could improve heart function.

Myocardial enzymes have been widely used as a biomarker for the clinical diagnosis of myocardial injuries (Yu and Wang 2018). When cardiomyocytes are inflamed (myocarditis) or necrotized (myocardial infarction) for various reasons, the enzymes contained in the cardiomyocytes can enter the blood, so the contents of these enzymes in the blood will increase. Myocardial enzymes such as LDH, CK and CK-MB are the most common biomarkers for myocardial ischemia and HF (Mallick and Januzzi 2015). After testing, it was found that HSY can significantly reduce the increase in CK, CK-MB, LDH and HYP caused by ISO. In order to further examine the myocardial injury, we performed HE staining to observe the morphology of rats in each group and Tunel staining to observe the apoptosis in the myocardium of rats in each group. The results of HE staining showed that there was a large amount of inflammatory infiltration in the myocardial tissue of the ISO group, the nucleus was dissolved or disappeared, and the arrangement of myocardial cells was disordered. Compared with the ISO group, inflammatory infiltration and a small amount of nuclear dissolution or disappearance were seen in the myocardial tissue of the HSY-L group, while the rat cardiomyocytes were arranged in an orderly manner with only partial inflammatory infiltration in the HSY-H group. Tunel staining results showed that HSY effectively reduced ISO-induced apoptosis in the myocardium of MF rats. Combined with myocardial enzymes, HE staining and Tunel staining results show that HSY has a protective effect on ISO-induced MF rat cardiomyocytes.

The Masson staining and immunohistochemical results showed that HSY can effectively reduce the collagen deposition in the myocardial tissue of rats with MF. PICP and ICTP are serum biochemical markers of cardiac ECM (Sun et al., 2017). These results showed that the ratio of PICP/ICTP in the ISO group was significantly higher than that in the control group. The PICP/ICTP ratio of the HSY groups was effectively decreased. The combined results of Masson and collagen I and III immunohistochemistry showed that HSY may reduce collagen deposition by regulating the rate of collagen production/degradation. In summary, these results showed that HSY could reduce the formation of collagen and increase its degradation, thereby reducing the deposition of collagen in myocardial tissue.

Previous studies have shown that Sirt3 may be a negative regulator of MF (Chen et al., 2015). Su et al. confirm that Sirt3 has a critical role in Ang-II-induced cardiac fibrosis and remodeling through pericyte transition and ROS-TGF-β pathway (Su et al., 2020). TGF-β has a strong inducing effect on the proliferation of ECM and plays a key role in the development of MF. TGF-β promotes phosphorylation of the Smad2, 3 complex by binding to the receptor (Dobaczewski et al., 2011). The complex form hetero-oligomeric complexes with Smad4 and enter the nucleus, promote the conversion of fibroblasts into myofibroblasts in the heart, and then increase collagen production (Xie et al., 2017). As an inhibitory Smad protein, Smad7 can bind to the activated type I receptors to inhibit signal transduction in the TGF-β family. The results of western blot indicated that HSY could increase the expression of SIRT3 and Smad7, inhibit the TGF-β/Smad signaling pathway, and suppress the conversion of fibroblasts to myofibroblasts and the production of collagen.

Matrix metalloproteinases (MMPs) are zinc-dependent endopeptidases that can degrade various ECM proteins (Laronha and Caldeira 2020). MMP activity can be inhibited by the molecular family of tissue inhibitors of metalloproteinases (TIMPs). Under physiological conditions, a dynamic balance is maintained between the MMPs/TIMP to maintain the ECM content. When MF occurs, this balance is disrupted. Through HSY intervention, the expression of MMP2, MMP9, and TIMP-1 tended to be normal. These results suggested that HSY could increase the degradation of collagen and reduce ECM deposition by regulating MMPs and TIMP-1.

In this study, we successfully established a rat model of MF induced by ISO. Compared to other animal models, the ISO-induced MF model was found to be more stable and more consistent with clinical pathology (Krenek et al., 2009). In addition, a total of 18 different kinds of compounds of HSY were identified using UPLC-ESIQ-Exactive Orbitrap/MS (Table 2). Synchronously, there was evidence that Calycosin-7-glucoside (He et al., 2013), Astragaloside IV(Wei et al., 2020), Calycosin-7-glucoside (He, Liu, Peng, Huang and Wu 2013), Schisandronic acid (Zhang et al., 2020) had inhibitory effects on MF, anti-inflammatory and anti-tumor activities. These may be the main effective ingredients in HSY to treat MF, and we will verify them in subsequent experiments.

Overall, we verified that HSY can protect myocardium, ameliorate ISO-induced MF, and improve cardiac function. This protective effect may occur in two ways. One is the activation of Sirt3, the inhibition of the TGF-β/Smad signaling pathway and prevent the transformation of fibroblasts into myofibroblasts, thereby inhibiting the production of collagen, and the other is to regulate MMPs, thereby increasing collagen degradation. In addition, in this study, we explored the blocking effect of HSY on ISO-induced MF, and the synergy between the main components of HSY will be carried out in subsequent studies.

## Data Availability

The raw data supporting the conclusions of this article will be made available by the authors, without undue reservation.
